# Implementing Mindfulness in the Mainstream: Making the Path by Walking It

**DOI:** 10.1007/s12671-016-0632-7

**Published:** 2016-10-19

**Authors:** Rebecca S. Crane

**Affiliations:** Centre for Mindfulness Research and Practice, School of Psychology, Bangor University, Bangor, LL57 2AS UK

**Keywords:** Mindfulness-based programs, Mindfulness-based stress reduction (MBSR), Mindfulness-based cognitive therapy (MBCT), Implementation, Intervention integrity, Teacher competence, Mainstream, Ethics

## Abstract

There is expanding interest in mindfulness-based programs (MBPs) within the mainstream. While there are research gaps, there is empirical evidence for these developments. Implementing new evidence into practice is always complex and difficult. Particular complexities and tensions arise when implementing MBPs in the mainstream. MBPs are emerging out of the confluence of different epistemologies—contemplative teaching and practice, and contemporary Western empiricism and culture. In the process of navigating implementation and integrity, and developing a professional practice context for this emerging field, the diverse influences within this confluence need careful attention and thought. Both contemplative practices, and mainstream institutions and professional practice have well-developed ethical understandings and integrity. MBPs aim to balance fidelity to both. This includes the need to further develop skillful expressions of the underpinning theoretical and philosophical framework for MBPs; to sensitively work with the boundary between mainstream and religious mindfulness; to develop organizational structures which support governance and collaboration; to investigate teacher training, supervision models, and teaching competence; to develop consensus on the ethical frameworks on which mainstream MBPs rests; and to build understanding and work skillfully with barriers to access to MBPs. It is equally important to attend to how these developments are conducted. This includes the need to align with values integral to mindfulness, and to hold longer-term intentions and directions, while taking small, deliberate steps in each moment. The MBP field needs to establish itself as a new professional field and stand on its own integrity.

## Introduction

There is expanding societal interest in mindfulness meditation. The precise reasons for this are not completely clear. We could hypothesize that it is an innate human need to have a practice to support connection and compassion around which we orient our daily life, and that in the movement away for some from organized religion there is a search to meet these needs in a new way. We could hypothesize that mindfulness offers a response to a time when people are looking for meaning and well-being in the midst of rapid change and challenge; perhaps a strong driving force may be the need to develop focus and cultivate peace in an increasingly frantic and competitive society; perhaps significant numbers of people seek mindfulness because it meets a particular need in their lives—to reconnect to themselves, and to connect with “bigger-than-self” concerns (Common Cause Foundation 2016).

What is clear is that we are in the midst of an emergent process of ancient contemplative practices becoming more and more an accepted part of mainstream life. We can see this as a confluence of rivers coming together—the epistemologies of contemplative teaching and meditative practice, and that of Western scientific method, medicine, and psychology merging to form something new. Each stream of understanding is interacting with, influencing, and enriching the other. However, there are some very real and challenging tensions inherent in the process of implementation of contemplative practices in the mainstream. How do we skillfully navigate the integration of paradigmatically different ways of approaching the experience of being human? Mindfulness meditation practices and teaching were not designed for clinical contexts, nor were they designed for implementation in Western mainstream institutions. They evolved as part of contemplative religious traditions to develop well-being and virtue (Davidson 2016). Critically, how do we ensure that the depth and integrity of these practices are maintained, and that they are offered in ways that ensure that their transformational potential is available to participants (Teasdale et al. 2003)? Conversely, how do we respect the pluralistic secular aspirations of our mainstream institutions as we support the translation and transition of mindfulness-based programs (MBPs) into new contexts?

Many have embraced the mindfulness popularity surge, but there are also some critical concerned voices (e.g., Segall 2013). While it is clear that the field is young, and that there are many unanswered questions and inevitable research gaps, there is little controversy that the empirical evidence is robust in some areas and promising in many others (Dimidjian and Segal 2015; Khoury et al. 2013). Broadly speaking, the critiques are of a different nature. On the one hand, there are concerns that mindfulness, when delivered outside of the frameworks for which it was originally developed, is vulnerable to becoming dissociated from its ethical foundations, and so becoming misappropriated for purposes for which it was not intended (Baer 2015; Harrington and Dunne 2015). This concern includes the potential risk that some MBPs and their teachers may not be paying enough attention to the systemic societal causes of human suffering (Forbes 2016). On the other hand, there is also concern that the practices are too closely linked with their originating Buddhist context and that presenting them as mainstream or secular is misleading (Monteiro et al. 2015). As Harrington and Dunne (2015, p. 262) expressed, “there is a risk that [this debate] could become increasingly entrenched and polarizing, in ways that will likely serve no one.”

From the perspective of direct engagement in training teachers to offer mindfulness-based stress reduction (MBSR; Kabat-Zinn 1990) and mindfulness-based cognitive therapy (MBCT) (Segal et al. 2013), engagement with policy makers in relation to mindfulness (Mindfulness All Party Parliamentary Group 2015), and supporting implementation of MBCT within the UK health service (Rycroft-Malone et al. 2014), this paper offers an examination of these tensions, an overview of current “on the ground” approaches to working with them, and some thoughts on moving forward with discernment. Underpinning the analysis is an intention to support integrity by drawing the best from the contribution that each discipline makes to MBPs.

## Tensions, Challenges, and Dilemmas

These are addressed under two broad headings—the multiplicity of meaning conveyed by the term “mindfulness” and then integrity from three perspectives—that of contemplative practices, of the needs and requirements of mainstream institutions, and of MBPs.

### Mindfulness: One Word, Many Meanings

Mindfulness has become a trend word conveying a diversity of understandings dependent on context. Its meaning spans a wide spectrum of activity and practice. These include meditation within faith-based Buddhist contexts; the integration of mindfulness within MBPs, such as MBSR and MBCT, taught in mainstream secular contexts involving careful, systematic build-up of mindfulness meditation over an intense 2-month training period (sometimes termed “first-generation” mindfulness-based interventions); “second generation” mindfulness-based interventions which make the Buddhist underpinnings explicit within the teaching process (Shonin and Van Gordon 2015); lower dose, lighter touch integration of mindfulness meditation into programs for the mainstream such as those used with schoolchildren or business people (Kuyken et al. 2013); and it also covers the current trend to add the word mindfulness as a prefix to an activity such as dog walking, coloring or knitting to convey an aim to conduct this activity quietly and peacefully. Politicians frequently insert the word mindful into their discourse to convey that they are being attentive and careful. This all creates a confusing context for the development of this work—conversations can take place using the same word while the parties are holding very different meanings.

**Fig. 1:**
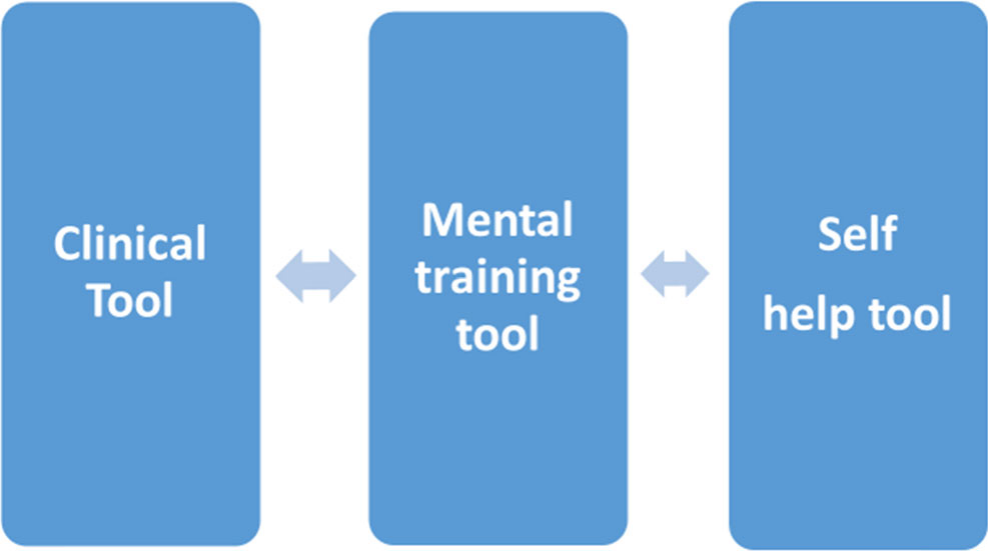
The spectrum of aims of mindfulness-based programs within mainstream settings

Even if we narrow our focus to the use of mindfulness within contemporary programs for secular mainstream settings, there is huge diversity in terms of the core aims of a particular program. MBPs are used as clinical tools (e.g., the use of MBCT for depression prevention), as mental training tools (e.g., the delivery of mindfulness in schools and workplaces), and as self-help/development tools (e.g., the take-up of mindfulness-based 8-week courses by the general public) (see Fig. 1). The development and shaping of program forms to meet the particular aims of learning for different populations within a different context is necessarily giving rise to a multiplicity of program forms.

**Fig. 2:**
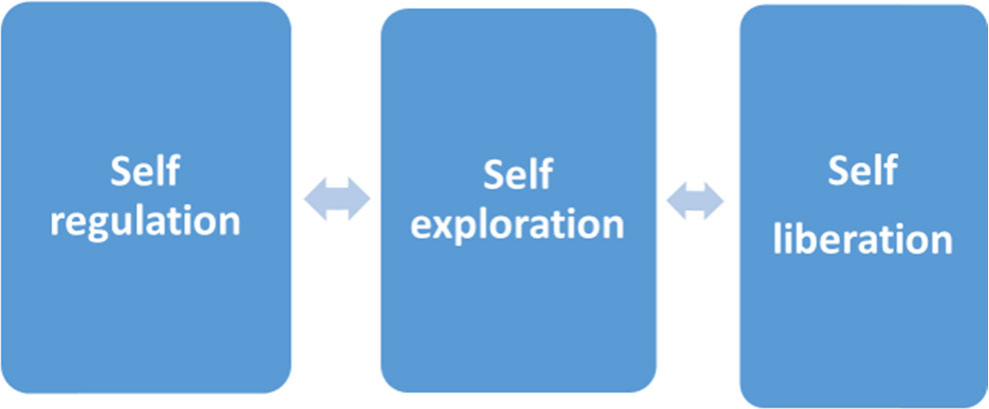
The spectrum on intentions and outcomes of MBP participants (drawn from Shapiro 1992). Self-regulation: i.e., a desire to work more skillfully with depression, stress, or relationships. Self-exploration: i.e., a desire to develop more skillful ways of relating to self and experience on a wider level. Self-liberation: i.e., a desire to engage in a deeper self and world exploration in ways which connect with “bigger than self” issues possibly motivated by a wish to be of compassionate service

Furthermore, there is never one straightforward aim when offering a mindfulness-based course. An MBP training process offers participants a wide variety of experiences: some take away a radical new orientation to their experience which spans the breadth of their life, while at the other end of the spectrum, others selectively take away a few strategies which they can apply to support stress reduction in their working life. Hence, the three aims highlighted in Fig. 1 can be in action in any one group in the range of different participants. The MBP teaching process is offered in an open-handed, invitational way that makes room for participants to connect with what speaks to them and leave aside what does not. Some of this is most likely pre-determined by the expectations the participants have when they arrive into the program, but many participants are surprised to discover themselves taking away unexpected learning and insights. Figure 2 highlights the spread of intentions that MBP participants start the course with and/or outcomes that they emerge from it with. Critically, given that the practices employed within MBPs do have the potential to cross these spectrum of effects, the teacher needs to have enough depth of personal experience with the practices and understanding of the processes at play, to enable her/him to relate to the depth to which participants might engage with the material.

Thus, it can be seen that the word mindfulness is pointing towards a breadth of meaning and activity. Therefore, any conversation about integrity, governance, and ethics needs to take particular account of the intentionality behind the delivery of a particular program, to a particular participant population, and in a particular context. For those within the MBP field, it is important that there is awareness of the instability of meaning of the word mindfulness and therefore of the need to use terms as accurately and clearly as possible. For example, using the term “mindfulness-based teacher” to refer to a mainstream MBP teacher to some extent distinguishes them from mindfulness teachers who offer teaching in Buddhist contexts and, when referring to a particular program form, use its full title, i.e., mindfulness-based stress reduction or MBSR.

### Integrity from Three Perspectives

#### Contemplative traditions and practices

“Everything rests on the tip of intention” (Feldman 2016). Clarity of intention of mindfulness-based program form and within the teacher delivering it are essential. In its fullest meaning, mindfulness is a radical reorientation to an individual’s approach to experience and to life. It embraces an understanding that it is inherently challenging to inhabit the human condition, and that suffering cannot be escaped but can be skillfully faced.

MBPs developed for delivery in mainstream contexts are naturally targeted at mainstream concerns (i.e., depression prevention, stress reduction) and are a time-limited short-term intervention (usually over 8 weeks). The motivating concerns and length of engagement in the training process are different from mindfulness practice within its originating religious contexts where the practices were used to enable the development of insight, wisdom, and virtue over an individual’s lifetime (Davidson 2016). However, there is of course much alignment between the underlying aspiration of mindfulness training to ease emotional distress and the societal need for this.

Tensions can arise if there is a divergence between the core ethical underpinning of mindfulness practice to do no harm and relieve emotional distress with that of institutionally driven and favored targets, such as hard work, high performance, and reduced absenteeism of maximization of profit. There are some risks and expressed concerns that the deeper transformative potential of the practice gets lost in the popularization of mindfulness in the mainstream as a way to create favored states such as calm and acceptance (Pursor and Loy 2013). Although a critique of mainstream mindfulness is that it risks developing passivity in the face of capitalism, in practice evidence suggests that individuals become more attuned to their own experiential process and empowered to make skillful choices in their life (Cook 2016). It is important though that MBP teachers and the wider field proactively engage with the societal and institutional issues which create collective distress. Some MBPs are explicitly intentioned to support individuals to change unsustainable institutional behaviors (Pykett et al. 2016), whereas others such as therapeutically oriented MBPs emphasize individual patterns. Teacher training requirements for these are somewhat different, but both sets of teachers need awareness of the wider cultural context within which human distress develops.

#### Mainstream institutions

Every institution, culture, region, and nation presents a context whose dynamics need understanding to enable successful implementation of a new approach within that setting (McCormack et al. 2002). A key to the success of the pioneering work of Kabat-Zinn (1990) in developing the MBSR was the way in which he created a program which balanced a number of potentially divergent issues: it skillfully met the challenges of people who were coming to the course; it honored the ethics, agenda, ethos, and concerns of the American mainstream hospital setting within which the course was implemented; and it maintained the rigor, integrity, and transformational potential of mindfulness practice. Multiple MBPs, which have developed out of the root form of MBSR, have built on this approach.

Over many years, our Western mainstream healthcare, educational settings, justice system, and workplaces have evolved their own forms of integrity. These include an ethos of accessibility to the breadth of the demographic of society, of public accountability for the sorts of activities which take place inside our institutions, of working in ways which serve and are in the interests of the general public who provide the funding for these institutions, of providing services which offer value for money, and of implementing practice which represents the best empirical evidence available (Horton 2006). It also includes professional codes of ethics for the range of professional activities that take place within these institutions. Within these overarching value systems, there will be a context-specific nuance for each service and setting. There is much to critique in the current context for public service in which arguably there is a gap between ethos and reality, and a shift from a wholehearted focus on service towards a new set of more individualistic values, beliefs, and institutional relationships. Nevertheless, the values surrounding the public service ethos are part of the founding principles of our institutions and are held dear by many working within them.

All this needs a depth of recognition and understanding when considering implementation of any new approach. MBPs, coming as they do with their own particular value systems, present particular challenges to implementers. Table 1 shows some of the places where MBP teachers might be challenged in terms of holding the value systems of both mindfulness and the institution within which they are operating side by side. One can see the importance from the perspective of the institution to prioritize service and evidence, and from the perspective of an MBP teacher to prioritize creating a process and “container” which enables a particular sort of investigation and learning to take place. Sometimes these priorities are challenging partners. However, these tensions require holding and inhabiting. They need skillful navigation rather than resolution. They point to some of the fundamental dilemmas that every human being experiences as they navigate through life, and therefore are grist for the mill for exploration within an MBP.

**Table 1:** Balancing fidelity to the ethos of MBPs and mainstream contexts

Ethos within MBP pedagogy	Mainstream institutional ethos
– Emphasis on process rather than outcome	– Goal orientation
	– Activity driven by targets
	– Measuring outcomes routinely to check efficacy
– Approaching internal and external experience non-judgmentally	– Emphasis on judgment and “view”
– Value placed on giving time and attention to the immediacy of the moment	– Emphasis on efficiency and productivity
– Emphasis on sensing experiencing	– Emphasis on conceptualization

#### Mindfulness-based programs

MBPs are the product of the integration of contemplative practices into the mainstream. They set out to balance fidelity to mainstream norms (i.e., religiously neutral, empirically tested, theoretically informed, ethically informed by professional context) and to the norms of mindfulness practice and teaching (i.e., teacher strongly embedded in personal experience of the practice, values led, emphasis on learning process rather than outcome). The point is not that these are incompatible but rather that the issues in both areas need valuing and attending to.

The emergence of MBPs is itself nested within wider developments within psychology, medicine, health care, and education which include other mindfulness-informed programs such as Acceptance and Commitment Therapy (Hayes et al. 2011), Compassion Focused Therapy (Gilbert 2009), Dialectical Behavioral Therapy (Linehan 1993), Mindful Self-Compassion (Neff and Germer 2013), and developments in the field of Positive Psychology (Seligman and Csikszentmihalyi 2000). Like MBPs, these also aim to change core values and approaches to life, and some involve engagement in a range of practices to support sustaining inner shifts beyond the end of the training; they share some underpinning theoretical ideas with MBPs, and many include mindfulness meditation practice in their approach. However, MBPs differ from these sister developments in one key way: they employ mindfulness meditation practice as a central foundational methodology. This fundamental feature of MBPs is important to the integrity of the approach because the entire theoretical basis and pedagogy rests upon the experiential engagement in meditation practice by both teacher and participant. It is hypothesized that through the teacher’s embodiment of the principles of mindfulness within the MBP teaching space, participants are enabled to begin experimenting with this different approach themselves. This feature, however, gives rise to some of the critical voices towards MBPs because it can seem as if there is an intention to transplant an entire practice and its accompanying ideological system out of its religious context, and into this MBP context.

A key tenet and ethic of MBPs has always been that it is important to recontextualize the Buddhist teachings into a form that is equivocally not Buddhist; is free of ideology, dogma, or religious references; and is universally accessible to people of all faiths and none. This has always been a delicate maneuver, and MBPs have found themselves caught in a cross current of divergent criticism—too Buddhist for some and not Buddhist enough for others. Some raise concerns that MBPs have a covert (Buddhist) agenda. Conversely, on the other side of the spectrum, some raise the concern that because MBPs are not sufficiently explicit about the Buddhist roots, the interventions do not offer a robust enough context for the teaching process. As a response to this, second-generation mindfulness programs are being developed, researched, and implemented which make the Buddhist underpinning explicit rather than implicit (Shonin et al. 2014; Singh et al. 2016). These will offer another choice to those seeking to train in mindfulness. However, for the reasons outlined in this paper, it is important that interventions such as MBSR and MBCT, which are designed for implementation in secular mainstream institutions, are held and delivered in ways that are religiously neutral. Of course, total neutrality is neither possible nor desirable. For example, the Christian culture within which the UK is situated has imbibed implicit Christian values. The key point is that the teacher and the curriculum is not overtly linked to any religion, and both have an intention of cultural and religious openness and humility (Hook et al. 2013).

The MBP teaching process is distinct and differs from traditional methods often employed in faith-based contexts. In an MBP teaching process, there is very little delivery of didactic teaching on ethics, virtue, or upfront teaching on the view or understanding which surrounds the meditation practices. The exception to this is within the first session in which the teacher facilitates participants in coming to agreement on ways of behaving within the group context, i.e., respecting each other’s contributions, confidentiality, and taking care of personal needs. The teacher then throughout is a custodian of the space, ensuring that the process is held ethically. As in many other therapeutic interventions, the ethics are mostly held implicitly. Participants are guided in meditation practices and group exercises, and are then invited to share and dialogue about what they noticed. The teacher (drawing on their implicitly held underpinning frameworks of understanding) supports the group to recognize key themes and understandings about the human mind/body as they emerge from the experience of the group. Participant experience leads—they are empowered to recognize their expertise in relation to their own experience, and make their own discoveries. Conceptual framing (if it happens) follows and is closely integrated with immediate experience. It is critically important that the integrity of this experientially led MBP teaching process is continued and maintained. It is through this that participants have the freedom to come to the course from a diversity of cultural, ethnic, and religious backgrounds with the confidence that their values and beliefs will be respected, and it is through this that participants are empowered to skillfully inhabit and honor their own process and experience. However, it is perhaps timely to make the particular philosophy and ethical process that informs and underpins MBP teaching more visible in contexts outside the container of an MBP teaching space.

Because the ethical basis, the value system, and the philosophical underpinnings to the programs are implicit rather than explicitly visible within the teaching process, the teacher takes quiet personal responsibility for holding the integrity of the process. There is a lot of unseen work taking place. The teacher is carrying frameworks of theoretical and practical understanding of the human mind, and of how these interface with the practice of mindfulness meditation. These are held in readiness so that they can be used to help participants make sense of experiential observations as they emerge. These frameworks are drawn from a range of settings—primarily from contemporary cognitive psychology, physiology, and aspects of Buddhist psychology. The teacher is also holding the ethical codes of their profession and of the institution. This is one reason why so much emphasis is placed on the teacher—they sit at the fulcrum point conveying the authenticity of the teachings, while also skillfully ensuring that the process is held and embodied in a context appropriate ethical framework.

## How is the MBP Field Navigating these Tensions Now and as it Moves Forward?

While MBPs need to continue to draw on and be informed by the disciplines which gave rise to them, it is important that they establish themselves as an independent field of inquiry, research and practice-based developments which can stand on their own integrity. Any newly emergent field has to work to find its own way of holding governance, standards, and ethics. As has been discussed, MBPs are navigating some unique sorts of tensions.

### What Do We Need to Do?

#### Continue to develop skillful expressions of the underpinning theoretical and philosophical framework for MBPs

As MBPs gain popularity in the mainstream, and are subject to critique and peer review, more attention is needed to proactively communicate the philosophical framework and intentions surrounding and underpinning them. We need to develop new clarity and new ways to language understandings to maximize accessibility. In part, this is about disseminating existing understanding—integrating and developing the frameworks of psychological understanding of the mind which draw from a range of sources including Buddhism and other wisdom traditions, and contemporary theories such as those of cognitive and neuroscience. Interestingly, some mindfulness teachers are increasingly broadening the conversation by investigating how MBPs draw on other philosophical traditions (e.g., Batchelor and Peacock 2016; Williams and Cullen 2016).

In the development of the field over the last 30 years, much work has taken place to articulate the underpinning theories and frameworks to MBPs. Empirical research on mechanisms is increasingly supporting the development of theoretical models and frameworks (e.g., Gu et al. 2015; Jha et al. 2010; van der Velden et al. 2015). Over time, these developments will support the field in refining understanding, and communicating clarity about the contribution mindfulness can make within the mainstream, and also the limits of its contribution.

#### Sensitively work with the boundary between mainstream mindfulness and religious mindfulness

Linked to the previous point, it is critical that MBPs designed for implementation with mainstream institutions are unequivocally contextualized within the norms of mainstream secular culture. It is important that the wisdom and frameworks of understanding from the contemplative traditions from which aspects of MBPs have been adapted continue to inform practice and thinking. However, the potential for mindfulness teaching and practice to speak to human experience across the demographic of society is at risk if this boundary is not sensitively managed. Because implementation is influenced by context, how this boundary is managed will vary within cultures, nations, and institutions. MBP teacher training processes must include training in sensitivity to these boundary issues between mainstream mindfulness and religious mindfulness. This is particularly critical for those teachers who do identify with a particular faith context and who draw support from that context for sustaining their own practice. This is not new. Many practitioners working in healing professions resource themselves personally within their own traditions and also need to skillfully translate their work so that it is accessible to those of all religions and none. This boundary definition and articulation is of course not entirely easy to do because every individual holds their own meaning around the domains of religion, spirituality, and secularity. However, the sensitivities to and concerns about the potential misuse of a position of power for ideological or religious indoctrination are real, and given that MBPs sit on a delicate edge between therapeutic practice and spirituality (Harrington and Dunne 2015), open dialogue is needed, and explicit ethical practice governance on this area for MBP teachers is required.

Linked to this issue, retreat opportunities for MBP teachers and course graduates are needed which are grounded in the depth of teachings underpinning mindfulness practice but are free of any religious context for the practice. This is increasingly happening. Retreat opportunities are being opened up which skillfully provide mindfulness teaching and practice for people who wish to frame their practice in a non-religious way. Many skilled and senior teachers who are deeply steeped in understanding of mindfulness teaching are recontextualizing the teaching for mainstream audiences and are choosing venues that minimize potential barriers to engagement. Through this, the vital work of supporting mindfulness practice communities to emerge is happening, which enable both MBP teachers and their course graduates to find a secular context for ongoing practice and inquiry.

#### Develop organizational structures within the mindfulness-based field which support governance and collaboration

Collaboration within the field on key issues that influence how the MBP field develops and in what form it is passed onto the next generation is essential. The integrity of the work is a shared responsibility for everyone engaged in it. As Kabat-Zinn (2011) said, “It has always felt to me that MBSR is at its healthiest and best when the responsibility to ensure its integrity, quality, and standards of practice is being carried by each MBSR instructor him or herself.” The teachers are the main conduits for the work. It is critical therefore that within training processes, there is a strong emphasis on developing self-regulation habits—reflective practice, supervision, personal mindfulness practice, understanding and working within current empirical evidence, and ensuring that teaching practice is seen and reviewed by peers and supervisors. However, lone teachers are limited in their capacity to implement if the wider context around them is unsupportive (Crane and Kuyken 2012). Mainstream contexts frequently include pressures for swift or low-cost implementation. It is tremendously supportive for grassroots teachers to be able to draw on the collective voice of leaders within the field who communicate consensual views on good practice and integrity.

Teacher training organizations have a particular responsibility to lead the development of the wider supportive professional context for mainstream mindfulness teachers. There are good models of effective networks of teacher training organizations working together to influence and govern professional practice for MBP teachers [e.g., European Associations of Mindfulness-based Approaches (EAMBA), 2014; Santorelli et al. 2011; UK Network for Mindfulness-Based Teacher Training Organisations, 2014]. These collaborations have led to influential good practice standards and ethical codes of conduct for teachers and trainers, to national policy influence, and to a national listing of teachers who have trained to minimum good practice levels and are adhering to ongoing good practice recommendations. An international integrity network is now forming to support the development of a coherent international voice on good practice issues. How these networks and others develop and organize themselves going forward is likely to determine how successful the field is in enabling dissemination of best practice and establishing credibility.

Current networks prioritize governance of MBSR, MBCT, and programs that have originated from these forms, and match them in terms of length of sessions and home practice. Collaborative work is needed by MBP teachers and trainers working in other contexts with other curriculums to develop and disseminate good practice standards. This is particularly needed in the context of the rapid implementation of MBPs in workplaces and educational settings.

#### Investigate teacher training, supervision models, and teaching competence

Over the last 15 years, work has taken place to build consensus and understanding about how best to train an MBP teacher (e.g., Crane et al. 2010; Marx et al. 2015), when someone is ready to teach an MBP, and when someone is ready to train others to teach MBPs (e.g., UK Network for Mindfulness-Based Teacher Training Organisations, 2015). Work has taken place and is underway to investigate teacher qualities and their links to participant outcome (Crane et al. 2013, 2016; Huijbers et al. under review). In the next phase of field development, particular and more research priority is needed on these questions because they are critical to enabling evidence to move successfully into practice (Dimidjian and Segal 2015). The body of evidence so far accumulated is appropriately weighted at the early stage of the research journey (Craig et al. 2008; Rounsaville et al. 2001). Energy and attention are now needed on the process investigations of how to enable the evidence to successfully embed in practice and what adaptations might be needed to enable accessibility (Dimidjian et al. 2014).

#### Develop consensus on the ethical frameworks on which mainstream MBP teaching rests

The ethical integrity of MBPs has been an issue of strong debate within the field (Baer 2015). Much of the tension resides in the dynamic created by the multiple influences on the development of MBPs, particularly the paradigm clash between the contemplative roots of mindfulness practice and the neoliberal culture of mainstream western life. These tensions arise particularly strongly in certain contexts such as the workplace, which on the one hand is less developed in terms of research and teaching practice governance, while on the other is developing at a rapid pace in terms of practice happening on the ground. Ethical tensions are real for all MBP teachers who tend to find themselves at the fulcrum of discovering skillful ways to translate their embodied experience of mindfulness practice into forms that are ethically and culturally consistent with the contexts within which they are implementing.

Baer (2015) cogently argued that MBPs for mainstream contexts should firmly ground ethical issues within long established and tested ethical codes for professional practice in these settings. Given that the primary intention of the overall project we are engaged in is to support accessibility to mindfulness training in the mainstream, this makes considerable sense. There is though the concern that some MBP teachers who have undertaken rigorous MBP training do not have a healthcare or educational professional practice within which to situate their MBP teaching because they have come to the work by other routes. This leaves these teachers and their participants somewhat unprotected and is an issue which needs addressing as we go forward.

Grossman (2015) reminded us that the “cultivation of mindfulness is inherently oriented toward the development of an ethical stance toward self, others and all animate and inanimate objects in the world” (p. 17). Hence, another key aspect of ethics in the context of MBPs is that as a teacher develops a personal practice, these inherent ethics become embodied within their teaching.

Ethics for MBP teaching can thus be expressed as having a dual emphasis on building embodied ethical integrity from the inside out (ensuring that teacher training involves thorough training and preparation which includes deep engagement with personal mindfulness practice, exploration of ethical issues, and examination of personal motivations), and from the outside in (ensuring that MBP work is framed within the context of established professional ethical codes of conduct and of the ethical structures embodied within mainstream public institutions). Work is needed to develop a consensual voice on these ethical issues and to embed them into teacher training processes.

#### Build understanding and working skillfully with the barriers to access to MBP training

Currently, the accessibility of MBP training to those at the margins of society is limited. The processes involved in expanding accessibility into hard to reach contexts are complex and nuanced. There are multiple barriers to engagement, many of which cross over with other therapeutic approaches. However, given the proximity of MBP teaching to personally held understandings about spirituality, religion, and secularity, the field has a particular responsibility to consider and develop sensitivity to the ways in which the practice of mindfulness may present barriers to engagement for some groups. New contexts need new skillful formulations and understandings. The basics of distress may be very similar, but the way they are experienced and the meanings held within them may be quite different. New cohorts of MBP teachers need to be supported and cultivated who are part of marginalized communities. All MBP teachers need support, training, and awareness building to enable them to more skillfully work in inclusive ways across the demographics of society.

### How Do We Need To Do It?

During the process of implementing MBPs, it is important to:

#### Align with values that are integral to mindfulness practice

Mindfulness training employs a methodology which enables the individual to (re)discover and (re)connect to their personally held deep frames and values. From this place of connection, the individual is more empowered to make choices that align with these values. These are held within a broad ethical intentionality to make choices to act in ways that are skillful to self and others. The process of implementing mindfulness needs to be similarly guided and informed by the values that are embodied within the practice of mindfulness. This includes dynamically inhabiting the tensions between seemingly opposite forces (i.e., the need for creativity within the field and the need for control and regulation; the importance of holding a wider vision for systemic societal change while engaging in worthwhile work within dysfunctional institutions), it includes holding an intention to take the long view by setting in place foundational building blocks for integrity within the field for the next generation, and it includes giving priority both to the process of implementing change as well as to the content of the change itself. It also includes a parallel emphasis on developing integrity from the inside out (training teachers so that there are expectations regarding on-going attention to self-integrity) and from the outside in (developing anchor points in the form of governance and standards that people from within and without the profession can relate to) (Crane and Reid 2016).

#### Hold a wider and longer-term intention and direction, while taking small, deliberate steps in each moment

Often what is possible for us are just small steps in the direction of a wider vision and aspiration we hold for ourselves and our world. However, developing and regularly reconnecting to wider intentionality is vitally important for informing the steps we take now.

There has been significant criticism of the MBP field for aligning too closely with and colluding with neoliberalism (Forbes 2016). Just as mindfulness practice supports the process of bringing into conscious awareness the individual’s habits of mind that drive behaviors, it is arguably important that as practice deepens, awareness of the wider social and contextual influences that drive collective behaviors are also brought into awareness. In this way, MBP teachers will become more sensitized to the nuances of how they situate their work; of how to recognize and work skillfully with gender, cultural, class, race, and power relations; and of how the work can be seen as one aspect of an integrated approach to building a sustainable world. Inquiry into these dimensions of the practice and the work needs to be built into training and supervision for MBP teachers going forward. Simultaneously, MBP teachers need to pragmatically meet the world as it is now. This includes engaging with policy makers and aligning the work to policy priorities, and it includes ensuring that developments are empirically informed.

## Conclusions

A new field is emerging, and it needs to stand on its own integrity. We are in the midst of an evolving integration between contemporary understandings about the conditions needed to support the human mind-body system to flourish and to skillfully work with distress, with that of ancient contemplative practices which offer a methodology for looking into the human mind and practically engaging with the processes therein. This is necessarily an emergent, live process. In its efforts to bridge and draw the best from the paradigms of Western scientific empiricism and that of centuries-old contemplative traditions, the process is inherently creative and tension filled.

There are two key principles that deserve particular emphasis. First, in all of these developments, it is critical to keep the interests of the general public at the center of our minds because this will keep us closely aligned with the originating intentionality of MBPs. Second, it is vital to focus on the quality of individual MBP teacher formation and the development of a supportive context for their practice. An MBP teacher in their early thirties might teach 4000 people in their career, so prioritizing the quality of training, supervision, and support this teacher receives has the potential to positively impact and touch a lot of people.

The project we are engaged in here is about finding skillful ways to bring contemplative practices into the mainstream. It is important to stay close to the values that guide this process. What do we really care about? How can MBPs positively influence individuals and society? The stance that MBPs take is that mindfulness-based practices can fruitfully stand independent of religion, but in order to do this, clarity is needed about the value system they rest upon and the intentionality that guides the direction of their engagement.
